# Late-onset depression predicts cognitive impairment and subsequent dementia among older adults with major depressive disorder: findings from UK Biobank and primary care linked data

**DOI:** 10.1192/bjo.2026.10995

**Published:** 2026-03-10

**Authors:** Lingfeng Xue, Mariia Bocharova, Allan H. Young, Dag Aarsland

**Affiliations:** Centre for Healthy Brain Ageing, King’s College London, UK; International Centre for Education and Research in Neuropsychiatry, Samara State Medical University of the Ministry of Health of the Russian Federat, Russian Federation; Centre for Affective Disorders, King’s College London, UK; Division of Psychiatry, Brian Sciences, Imperial College London, UK; Centre for Age-Related Medicine, Stavanger University Hospital, Norway

**Keywords:** Depressive disorders, dementias/neurodegenerative diseases, longitudinal data, old age psychiatry, survival analysis

## Abstract

**Background:**

Late-life depression (LLD) is associated with cognitive impairment and an elevated risk of dementia, yet the influence of age at depression onset on cognitive prognosis remains unclear. Emerging evidence suggests that late-onset depression, defined as first depressive episode in later adulthood, may reflect distinct neuropathological mechanisms and predict more severe cognitive decline and greater dementia risk than early-onset depression.

**Aims:**

This study aimed to investigate whether late-onset depression is linked to domain-specific cognitive impairment and higher risk of incident dementia among older adults with major depressive disorder.

**Method:**

We analysed UK Biobank data from older adults (aged ≥60 years) with primary care linkage, classifying participants into depression-free controls, early-life depression, late-life depression with early onset (LLD-EO) and late-life depression with late onset (LLD-LO). Cognitive performance across these five domains was assessed cross-sectionally at baseline using touchscreen tasks. Incident dementia was evaluated prospectively using clinical records up to 2022. Multi-level models with inverse-probability weighting and survey-adjusted mixed modelling were applied to assess group differences in cognitive function, controlling for demographic covariates, lifestyle factors and physical and mental health conditions. A Cox regression model was employed to estimate dementia risk among groups.

**Results:**

Among 75 064 participants aged ≥60 years, the LLD-LO group (*n* = 4858) showed significantly worse cognitive performance than healthy controls, particularly on fluid intelligence and visuospatial memory. The LLD-LO group performed worse than LLD-EO on fluid intelligence. During follow-up, LLD-LO was associated with a higher risk of incident dementia (hazard ratio 1.42–1.52) across all adjusted models. Deficits in fluid intelligence and visuospatial memory partially mediated the link between LLD-LO and subsequent dementia.

**Conclusions:**

Late-onset depression showed more severe impairment in fluid intelligence compared with LLD-EO. Late-onset depression was associated with increased incident dementia compared with depression-free individuals.

Late-life depression (LLD) poses significant challenges to geriatric health and life quality. LLD is normally defined as a diagnosis of major depressive disorder (MDD) in older people, and affects approximately 7% of adults aged 60 years and above.^1^ LLD is associated with multiple geriatric conditions and elevated mortality, and also with cognitive impairment in various domains including episodic memory, information-processing, executive functioning and visuospatial ability.^2,3^ Importantly, depression is an established risk factor for dementia, which affects 5–7% of community-based individuals aged 60 years and over.^4,5^ Dementia is a major burden for late-life healthcare,^6^ and the relationship between LLD and dementia is complex. Depression may represent an early manifestation of dementia with shared pathological mechanisms, or act as an independent aetiological risk factor.^5^ Evidence from neuroimaging and neuropathological studies supports overlapping correlates, including vascular burden, elevated neuroinflammation, neurotrophic abnormalities, amyloid accumulation and hippocampal atrophy,^7^ underscoring the potential bidirectional relationship between LLD and dementia. Age at onset of first depressive episode has gained increasing attention regarding cognitive decline and progression to dementia. Previous research has suggested that late-onset depression (LOD) may differ biologically and cognitively from early-onset depression (EOD).^8^ Specifically, LOD may be associated more with vascular burden and neurodegenerative changes, which could contribute to a more severe cognitive decline; for instance, hippocampal changes in LOD were associated with increased risk for mild cognitive impairment.^9^ Despite this, there is no consensus on whether it is specifically LOD that increases dementia risk. Whereas some studies showed that LOD may reflect early manifestations of underlying neurodegeneration,^10^ others showed that risk of dementia is elevated in both early- and midlife-onset depression.^11^ Moreover, accumulating evidence suggests that the trajectory of depressive symptoms, especially increasing depressive symptoms in later life, represents the highest risk of dementia incidence.^12^ The present study aimed to explore the association between age at onset of depression and cognitive impairment and dementia risk in older people with MDD. Specifically, we hypothesised that LOD would show greater cognitive impairment in various domains compared with depression-free older people and individuals with EOD, and that LOD is associated more with an increased risk of incident dementia.

## Method

### Design and participants

This study employed both a groupwise cross-sectional and a prospective longitudinal design. It utilised data from UK Biobank (UKB), a nationwide, population-based prospective cohort of >500 000 adults, between 2006 and 2010. For the present study, baseline was defined as the date of assessment centre attendance. Participants were followed up with surveys on various psychological, functional and lifestyle parameters until 2022; the median of the follow-up period is 13.69 years. In addition, UKB also included primary care linked data, which detailed clinical events, prescriptions and registration records with GPs. Primary care linked data covered approximately 45% (around 230 000 participants) of the UKB cohort. The present study includes all clinical data starting from 1990. Details regarding UKB and primary care linked data can be found in Supplementary Material, Appendix 1 available at https://doi.org/10.1192/bjo.2026.10995.

Participants in UKB with primary care linked data were included if they (a) were aged 60 years or older, (b) attended an assessment centre at baseline, with completion of at least one cognitive task, and (c) had primary care linked data in UKB. Participants were excluded if (a) they had a record of dementia before or within 1 year following the assessment centre visit; (b) they had a record of end-stage renal disease, motor neuron disease, stroke or all-cause Parkinsonism; (c) they had a record of major psychiatric conditions, including Parkinsonism, organic mental disorders, delirium, mental disorders due to physical disease, substance use-related disorders, schizophrenia and psychotic disorders, or bipolar affective disorders; (d) their record of dementia diagnosis lacked specific recording of dates; and (e) their primary care linked data suggested treatment-resistant depression, which was assessed using the definition developed by Fabbri et al for UKB primary care linked data.^13^ This research was conducted using data from the UKB resource under project no. 82087, which operates under Research Tissue Bank approval from the North West Multi-Centre Research Ethics Committee (REC reference no. 21/NW/0157). All participants provided written informed consent at the time of recruitment for the anonymous use of their data. Details and numbers of participants under each inclusion or exclusion category can be found in Supplementary Fig. 1.

### Diagnosis of depression and lifetime episodes

Diagnosis of depression was based on either ICD-9 and ICD-10 criteria or textual description in the clinical events record of primary care linked data.^14,15^ Codes 296.2 and 296.3 were identified in ICD-9 as a depression diagnosis, as well as codes F32 and F33. Records of depressive symptoms (e.g. low mood, loss of interest, hopelessness, etc.) were not recognised as a diagnosis of depression. Diagnostic records without valid dates were excluded from the analysis. All target records were searched using Read codes, a coded thesaurus of clinical terms in primary care (see Supplementary Table 1 for details). Consecutive records within an 8-week period were considered a single episode, unless they were separated by remission records. The present analysis adopted age 50 years as the threshold for late-life depressive episodes, because it was considered inclusive for age-at-onset cut-off.^8^ Based on records of lifetime episodes, participants were classified into either (a) the no-depression group, if they had no lifetime depressive episodes (healthy controls); (b) the early-life depression (ELD) group, if all episodes occurred before the age of 50 years; (c) the LLD with early onset (LLD-EO) group, if their episodes started before age 50 years (i.e. early-life episode) and ended after age 50 (i.e. late-life episode); and (d) the LLD with late onset (LLD-LO) group, if all episodes started after age 50 years. Grouping and numbers of participants can be found in Supplementary Fig. 1, and a diagram of grouping in Supplementary Fig. 2.

### Measures and dementia incidence

The primary outcome of groupwise cross-sectional analysis was the performance of the five cognitive tasks administered at baseline visit. These tasks evaluated the ability of reaction time, fluid intelligence (verbal reasoning), numeric memory, pairs-matching (visuospatial memory) and prospective memory. The reaction time test assessed processing speed by measuring the average time taken to press a button when matching visual symbols appeared. The fluid intelligence test assessed reasoning ability by counting the number of logic and problem-solving questions answered correctly within a 2 min time limit. The pairs-matching test evaluated visuospatial memory by recording the number of errors made when recalling the positions of matching symbol pairs across two card-based rounds. The numeric memory test measured working memory span by identifying the maximum length of digit sequences that participants could correctly recall. The prospective memory test assessed delayed intention recall by testing whether participants remembered to disobey a later instruction based on an earlier cue. Details of the process and scoring of the five tasks can be found in Supplementary Material, Appendix 2. These tasks showed good reliability and validity in a previous validation study.^16^ Participants completed the tasks using touchscreen monitors and without supervision.

Incidence of dementia was reported as the primary longitudinal outcome. Dementia records were extracted from various health outcome fields in UKB, sourced from both clinical records and self-report events following a previous study.^17^ All-cause dementia, including Alzheimer’s disease, vascular dementia and unspecified other dementia, was considered.

To consider the effect of mood status on baseline cognitive tasks, Patient Health Questionnaire 2 (PHQ-2) was administered, a screening tool valid for major depression in older people.^18^ Demographic information including age, gender, body mass index (BMI) and education level were acquired, as well as the Townsend deprivation index (TDI), an estimate of social deprivation level based on various factors including employment, income, housing and living environment. Physical activity was measured by summed metabolic equivalent task (MET) minutes per week, which represent the energy cost of all forms of physical activities. Education level was dichotomised by the presence or absence of higher education. For lifestyle measures, smoking and alcohol use status were further included. Smoking status was dichotomised by experience of smoking, and alcohol use was recorded using a six-point Likert scale to measure drinking frequency. Physical diseases were also considered based on a history of heart and vascular conditions (heart attack, angina, stroke and hypertension), diabetes and cancer, which were reported by participants at the assessment centre and follow-up visits. History of receiving antidepressant treatment (both pharmacological and non-pharmacological) was also included.

### Statistical analysis

Cognitive task outcomes were standardised. To maintain normality, the outcome of pairs-matching (number of incorrect matches) was log transformed. A cognitive composite score was calculated using principal component analysis (PCA), and is represented by the coordinate of the individual along the first principal component.

Baseline group comparisons used analysis of variance (with Bonferroni *post hoc* tests) for continuous variables and chi-square tests for categorical variables. To address group imbalance, inverse-probability weighting (IPW) was applied using propensity scores from multinomial logistic models based on baseline characteristics. Cognitive outcomes were analysed using survey-weighted, generalised linear mixed models. Models for each cognitive measure as an outcome were constructed in two steps. First, three groups with depression history were regarded as separate multinominal levels and were compared with the healthy control group as baseline reference. Then, comparisons were conducted pairwise between all groups with Bonferroni correction for multi-testing. The analyses adopted a multi-level modelling approach to better account for demographics, psychological status and lifestyle and physical conditions. Level 0 modelling was unadjusted. For level 1 modelling, age and gender were included as covariates. For level 2 modelling, BMI, PHQ-2 score, education, MET and TDI were further included. For level 3 modelling, smoking and alcohol use status, physical condition and history of depression treatment were additionally included. Propensity scores were calculated accordingly for each level based on the inclusion of age, gender, BMI, education, MET, TDI, smoking and alcohol use status for IPW. Cohen’s *d* and odds ratios with 95% confidence intervals were calculated for group comparisons. Incident dementia was predicted with Cox regression analysis, using incidence of dementia and time to dementia as outcomes. Participants with depressive episodes during follow-up were excluded, to preserve baseline grouping validity. Models used the same multi-level adjustments (levels 0–3), and hazard ratios with 95% confidence intervals were reported. Pairwise hazard ratio comparisons applied Bonferroni correction as the next step. To account for death as a competing event against dementia, the Fine–Gray competing risks model was further employed, with full adjustment. Causal mediation assessed the indirect effect of baseline cognitive deficits on dementia risk (LLD-LO versus healthy controls), using fully adjusted logistic regression in place of Cox regression due to model constraints, because time-to-event data were not supported in such modelling.

All analyses were conducted using R version 4.4.1 for Windows (https://www.r-project.org/), with the ‘survey’,^19^ ‘survival’^20^ and ‘mediation’ packages for various analyses.^21^

## Results

A total of 75 064 participants were included in the study. Based on history of depression, 66 537 were grouped into healthy controls, 2559 into ELD, 1110 into LLD-EO and 4858 into LLD-LO. Table 1 shows the baseline characteristics across groups. There were significant differences among groups regarding most demographic and clinical measures (see Supplementary Table 2 for details), which validated the necessity of implementing IPW for all group-wise comparison models. Propensity scores for all modelling levels demonstrated adequate group balancing (see Supplementary Table 3 for details).

**Table 1:** Baseline characteristics across study groups

Variables	No depression	Early-life depression	Late-life depression	
			Early onset	Late onset
*n*	66 537	2559	1110	4858
Age, mean (s.d.)	64.55 (2.85)	63.97 (2.76)	63.90 (2.71)	64.58 (2.84)
Gender, male, *n* (%)	30 267 (45.49)	741 (28.96)	267 (24.05)	1604 (33.02)
BMI, mean (s.d.)	27.46 (4.37)	27.73 (4.80)	28.21 (5.15)	28.15 (4.70)
Education, college, *n* (%)	18 258 (37.28)	720 (38.69)	295 (36.92)	1093 (33.18)
TDI, mean (s.d.)	−1.79 (2.77)	−1.69 (2.80)	−1.53 (2.95)	−1.54 (2.89)
MET, mean (s.d.)	7.50 (1.04)	7.44 (1.07)	7.44 (1.10)	7.47 (1.07)
Smoking, smokers, *n* (%)	28 661 (43.28)	1125 (44.10)	478 (43.18)	2211 (45.71)
Alcohol use,^a^ mean (s.d.)	2.90 (1.54)	3.08 (1.61)	3.31 (1.63)	3.20 (1.59)
Physical diseases, *n* (%)	14 604 (21.95)	268 (24.14)	574 (22.43)	1254 (25.81)
PHQ-2, mean (s.d.)	0.33 (0.82)	0.63 (1.11)	1.20 (1.50)	0.90 (1.32)
Age at first onset, mean (s.d.)	–	37.62 (9.37)	39.39 (9.26)	57.30 (4.48)
Age at last episode, mean (s.d.)	–	38.76 (9.11)	58.00 (4.55)	58.61 (4.59)
Multiple episodes, *n* (%)	–	312 (12.19)	1101 (99.19)	1272 (26.18)
Dementia incidence, *n* (%)	1255 (1.89)	52 (2.03)	30 (2.70)	136 (2.80)
AD, *n* (%)^b^	682 (58.34)	27 (51.92)	16 (53.33)	76 (55.88)
VD, *n* (%)	189 (15.06)	8 (15.38)	6 (20.00)	20 (14.71)
UOD, *n* (%)	384 (30.60)	17 (32.69)	8 (26.67)	40 (29.41)

BMI, body mass index; TDI, Townsend deprivation index; MET, metabolic equivalent task score; PHQ-2, Patient Health Questionnaire 2; AD, Alzheimer’s disease; VD, vascular dementia; UOD, other or unspecified dementia.

a. Alcohol use was recorded using a 6-point Likert scale regarding alcohol intake frequency, with 1 for ‘never’ and 6 for ‘daily or almost daily’.

b. Percentages of dementia types were calculated from all dementia cases within each group (rather than percentages of total cases).

### Performance on cognitive domains at baseline across age-at-onset groups

**Fig. 1:**
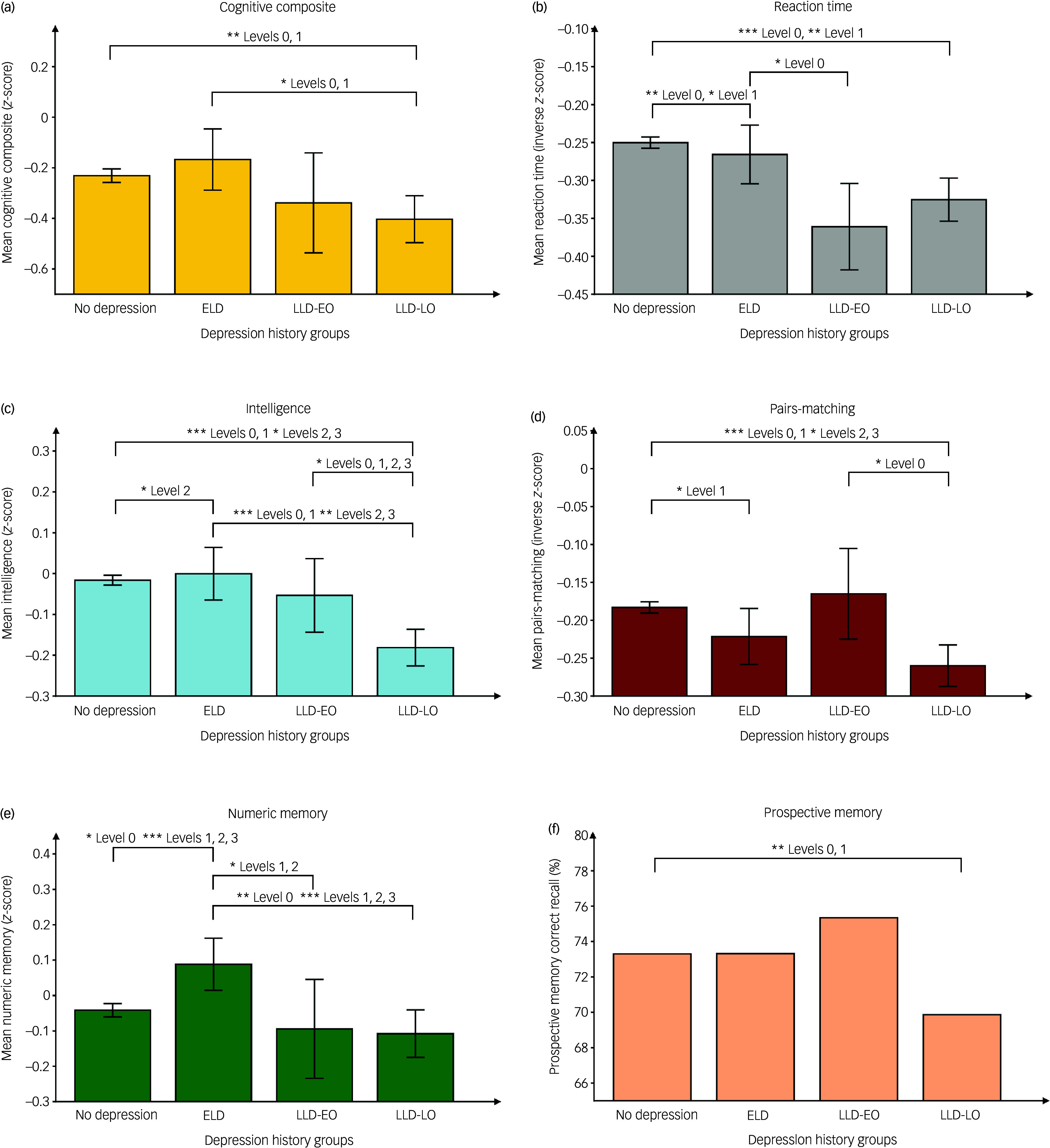
(a–f) Pairwise comparison of cognitive performance across depression history groups. *P < 0.05, **P < 0.01, ***P < 0.001. Bonferroni correction was applied in all pairwise comparisons. ELD, early-life depression; LLD-EO, late-life depression with early onset; LLD-LO, late-life depression with late onset. Levels of adjustment: level 0, unadjusted; level 1, adjusted for age and gender; level 2, adjusted for age, gender, body mass index (BMI), Patient Health Questionnaire 2 (PHQ-2) score, education and Townsend deprivation index (TDI); level 3, adjusted for age, gender, BMI, PHQ-2 score, education, TDI, smoking and alcohol use status.

Baseline cognitive performance was detected using a survey-weighted, generalised, linear mixed model. PCA suggested that cognitive composite score would provide acceptable representativeness for cognitive functioning, explaining 36.47% of the overall variance (see Supplementary Table 4). Results, with full adjustment using healthy controls as baseline reference, are shown in Table 2, and *post hoc* pairwise comparisons are illustrated in Fig. 1. Compared with the healthy controls group, the LLD-LO group showed inferior performance on cognitive composite score at level 0 and 1 modelling, but not following full adjustment (Cohen’s *d* = 0.13, 95% CI [0.05, 0.21]). No significant differences were found regarding reaction time following full adjustment. Regarding fluid intelligence, LLD-LO showed worse performance than healthy controls at all levels (Cohen’s *d* = 0.17, 95% CI [0.12, 0.22]). ELD showed better performance than healthy controls at level 2 (Cohen’s *d* = 0.02, 95% CI [−0.05, 0.08]). *Post hoc* pairwise comparison also showed that LLD-LO had worse performance than LLD-EO at all levels (Cohen’s *d* = 0.13, 95% CI [0.03, 0.24]), and worse performance than ELD at all levels (Cohen’s *d* = 0.19, 95% CI [0.11, 0.27]). Pairs-matching performance, indicating visuospatial memory, was found to be inferior in the LLD-LO group compared with healthy controls at all levels (Cohen’s *d* = 0.08, 95% CI [0.05, 0.11]). By contrast, ELD showed better performance than healthy controls at levels 0 and 1 (Cohen’s *d* = 0.04, 95% CI [0, 0.08]). Regarding numeric memory, ELD showed better performance than healthy controls at all levels (Cohen’s *d* = 0.14, 95% CI [0.04, 0.23]). LLD-EO showed worse performance than healthy controls following full adjustment (Cohen’s *d* = 0.05, 95% CI [−0.09, 0.21]). ELD was further found to have better performance than LLD-LO at all levels in the *post hoc* analysis (Cohen’s *d* = 0.21, 95% CI [0.09, 0.33]). Regarding prospective memory, LLD-EO exhibited better performance than healthy controls following full adjustment (odds ratio 1.11, 95% CI [0.89, 1.39]). Detailed results, with all levels of adjustment, can be found in Supplementary Table 5.

**Table 2:** Comparison of baseline cognitive performance with HC as reference, using survey-weighted, generalised, linear mixed modelling with full adjustment (mean and standard error of coefficients)

Variables	Cognitive composite	Reaction time	Fluid intelligence	Pairs-matching	Numeric memory	Prospective memory
Depression history groups						
ELD	0.10 (0.08)	−0.01 (0.03)	0.07 (0.04)	0 (0.03)	0.18*** (0.04)	−0.10 (0.12)
LLD-EO	−0.11 (0.08)	0.08 (0.05)	0.06 (0.07)	0.02 (0.05)	0.01 (0.08)	0.34 (0.23)
LLD-LO	−0.11 (0.08)	0.01 (0.03)	−0.09^*^ (0.04)	0.05^*^ (0.02)	−0.03 (0.05)	−0.20 (0.12)
Covariates						
Age	−0.03** (0.01)	0.04*** (0)	−0.02^*^ (0.01)	0.03*** (0.01)	−0.00 (0.01)	−0.02 (0.02)
Gender	0.26** (0.08)	−0.22*** (0.03)	0.11^*^ (0.05)	0.02 (0.03)	0.14** (0.05)	0.07 (0.12)
PHQ-2 score	−0.06 (0.04)	0.03 (0.01)	−0.04^*^ (0.02)	0.02 (0.02)	−0.02 (0.02)	−0.09 (0.05)
BMI	−0.02 (0.01)	−0 (0)	−0.01 (0)	−0.01^*^ (0)	−0.01** (0.01)	−0.01 (0.01)
TDI	−0.01 (0.02)	0.01** (0)	−0.01 (0.01)	0.01 (0.01)	−0 (0.01)	−0.02 (0.02)
MET	−0.12*** (0.04)	0 (0.01)	−0.11*** (0.02)	0 (0.02)	−0.06^*^ (0.02)	−0.16** (0.06)
Education	0.45*** (0.09)	−0.05 (0.03)	0.47*** (0.04)	−0.07^*^ (0.03)	0.16*** (0.05)	0.31^*^ (0.12)
Alcohol	−0.07** (0.02)	0.02^*^ (0.01)	−0.05*** (0.01)	0 (0.01)	−0.03^*^ (0.02)	−0.10** (0.04)
Smoking	0.06 (0.08)	−0 (0.03)	0.01 (0.04)	−0.10** (0.03)	−0.04 (0.05)	0.26^*^ (0.12)
Physical diseases	0.02 (0.09)	0.02 (0.03)	−0.01 (0.05)	−0 (0.03)	0.07 (0.05)	−0.15 (0.14)
Depression treatment	0.04 (0.09)	0.02 (0.03)	0.02 (0.05)	0.04 (0.04)	0 (0.06)	0.13 (0.14)

HC, healthy controls; ELD, early-life depression; LLD-EO, late-life depression with early onset; LLD-LO, late-life depression with late onset; PHQ-2, Patient Health Questionnaire 2; BMI, body mass index; TDI, Townsend deprivation index; MET, metabolic equivalent task score

All groups with depression history were compared with depression-free HC as baseline reference.

Levels of adjustment: level 0, unadjusted; level 1, adjusted for age and gender; level 2, adjusted for age, gender, BMI, PHQ-2 score, education, MET and TDI; level 3, adjusted for age, gender, BMI, PHQ-2 score, education, MET, TDI, smoking, alcohol use status, physical diseases and depression treatment. All coefficients with statistical signifiance (*P* < 0.05) were denoted in bold font.

* *P* < 0.05, **P < 0.01, ***P < 0.001.

### Age at onset of depression predicting dementia incidence during follow-up

**Fig. 2:**
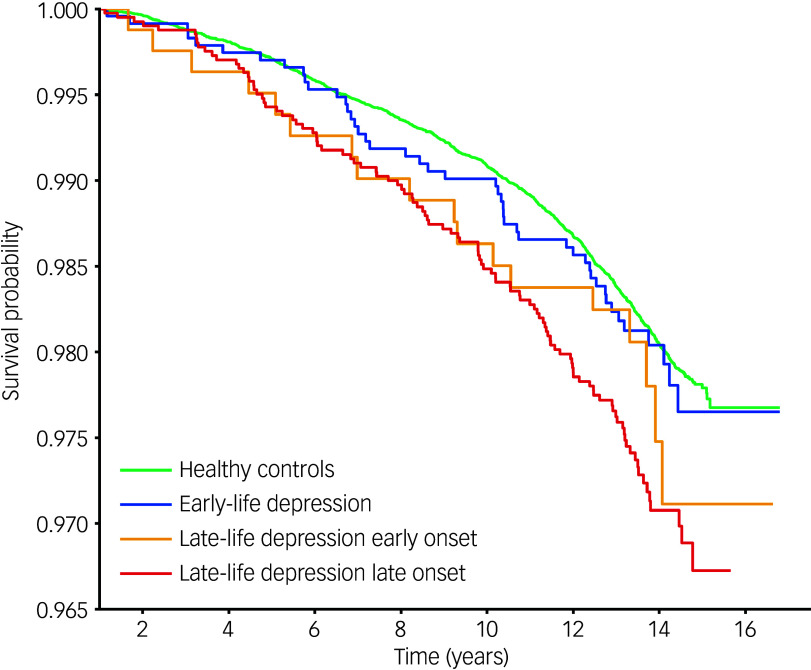
Kaplan–Meier survival curves for depression history groups predicting dementia (with fully adjusted modelling).

A total of 2590 participants were excluded because they developed at least one depressive episode during follow-up; the median of the follow-up period was 13.69 years (mean follow-up period 13.34 years). Among the remaining 72 474 participants, 1473 developed dementia. Cox regression was employed to investigate the risk of dementia for depression history groups over time. Propensity scores for all Cox regression modelling levels demonstrated adequate group balancing (see Supplementary Table 6). Cox regression revealed that LLD-LO predicted elevated risk for dementia compared with healthy controls at all levels of modelling, with a hazard ratio of 1.41–1.52 (see Supplementary Table 7 for details). *Post hoc* pairwise comparison for relative hazard ratio among groups supported the elevated risk of LLD-LO compared with healthy controls at levels 0 and 1. Additionally, LLD-EO predicted elevated risk compared with healthy controls only at level 1, which was not supported by *post hoc* pairwise comparison. No significant hazard ratio was found among other group pairs following correction for multi-testing (see Supplementary Table 8). Kaplan–Meier survival curves indicated that the LLD-LO group had a steeper decline in dementia-free survival compared with healthy controls over the follow-up period (see Fig. 2 for fully adjusted modelling curves). The Fine-Gray model, using death as the competing outcome, further confirmed that only LLD-LO predicted elevated risk for dementia compared with healthy controls (hazard ratio 1.46, 95% CI [1.06, 2.00]). Furthermore, mediation analysis with fully adjusted modelling was conducted between LLD-LO and healthy controls groups only (*n* = 50 433), with both fluid intelligence and pairs-matching included as mediators (because these are cognitive domains, with a significant difference between healthy controls and LLD-LO at all modelling levels). Results suggested that impaired fluid intelligence mediated 3.1% (95% CI [1.2%, 5.0%]) between LLD-LO and dementia incidence, with pairs-matching mediating 1.4% (95% CI [0.4%, 2.5%]; details can be found in Supplementary Table 9).

## Discussion

The present study, which included nearly 75 000 people aged over 60 years, investigated the effect of age at onset in LLD on cognitive functioning and the risk of dementia. We found that LOD showed inferior performance in various cognitive domains, especially regarding verbal reasoning and visuospatial memory, compared with depression-free individuals. Moreover, LOD presented inferior performance in verbal reasoning compared with LLD-EO. Cox regression further showed that LOD presented a higher risk for incident dementia than depression-free individuals, which was partially mediated by fluid intelligence and visuospatial memory.

A key feature of this study is its differentiation between early- and late-onset depression, highlighting the potential significance of a late-life onset of depressive episode as first-time depression onset in relation to cognitive outcomes. However, previous findings appear to be contradict the theory that the cumulative depressive course contributes to cognitive burden,^22^ because LOD tended to be linked with fewer episodes in this study and others.^23^ Previous studies found that LOD showed worse performance than EOD in multiple cognitive domains, including memory, visuospatial abilities, verbal fluency, reasoning and verbal learning.^24^ Combined with our findings, it is implied that LOD might be heterogenous from LLD starting in early or midlife in terms of cognitive profile and its neurological correlates. An earlier study using magnetic resonance imaging suggested that LOD had more pronounced volume reduction in some hippocampal regions than EOD, and that there was a significant correlation between cognitive performance and hippocampal volume in the LOD group.^25^ This was supported by a study that monitored cerebral blood flow (CBF) in remitted LLD, which suggested that LOD was associated with greater CBF abnormalities than EOD and that abnormal CBF was correlated with cognitive impairment in LOD.^26^ Moreover, white matter lesions (WML) may be associated with cognitive decline in LOD; previous studies have suggested that WML is more severe in LOD than in EOD.^27^ Lesion burden in several subcortical regions was found to be correlated with poor cognitive performance, and that these regions were critical in regard to complex thinking skills.^28^ These findings could account for cognitive impairment in LOD as compared with EOD in this study.

This study also found an elevated risk of subsequent dementia in the LLD-LO group compared with healthy individuals. Interestingly, previous studies reported that cumulative lifetime episodes might be linked with incident dementia. For instance, it was demonstrated that individuals with mid- to late-life depressive symptoms had an 80% increased risk of developing dementia, whereas those with only late-onset symptoms had a 70% increased risk.^11^ However, other studies have suggested that LOD poses a higher risk of dementia than EOD, although the former lost significance following control for various medical and lifestyle factors.^29^ One explanation for differential results could be the lower threshold of age at onset applied in this study. Brain changes in disorders leading to dementia may occur 15–20 years before the onset of clinical features,^30^ and thus defining late onset as beyond age 60 years and applying the inclusion criterion of age >60 at the same time might fail to encompass all cases of depression prodromal to dementia within the late-onset group. Our study showed that, when the age threshold was set at 50 years, LOD emerged as a strong predictor of dementia and exhibited more cognitive decline compared with earlier-onset cases. However, a precise and clinically meaningful age threshold still requires further investigation. In addition, although LLD-LO showed a statistically significant elevation in dementia risk compared with controls, whereas LLD-EO did not, *post hoc* contrasts indicated that the hazard ratios for both groups were numerically similar and not significantly different, suggesting that any direct difference between the two groups is modest and should be interpreted with caution.

Meanwhile, several risk factors could contribute to the elevated risk of dementia in LOD, rather than EOD. Vascular risk was found to be associated with cognitive impairment in LOD versus EOD,^31^ which could influence the future risk of dementia, especially in vascular and mixed types.^32^ Apart from this, depression can be prodromal to neurodegenerative diseases. This was summarised in a review that included epidemiological, pathological and biomarker perspectives,^33^ and recent findings have suggested a high proportion of LLD cases transitioning to dementia (13%), which was associated with later age at onset.^34^ These findings indicate that neurodegenerative disease could manifest as depressive symptoms recognised as LOD in the early stages. Apart from age at onset, both the timing and pattern of depressive symptom emergence may also be important as risk factors for dementia. Evidence from trajectory studies further suggests that late onset of depressive symptoms is linked to vascular burden, white matter hyperintensity and poorer executive function, even when compared with consistently high-symptom groups.^35^ This is supported by another study which found that increasing symptom trajectories in midlife were associated with greater white matter abnormalities, with a steady high-symptom scenario being linked to poorer cognition and reduced medial temporal lobe volume.^36^ Notably, these studies used repeated-depression scales and cognitive assessments, which may yield different subgroup classifications and results compared with our approach based on lifetime clinical diagnosis records. Moreover, the differential predictability between EOD and LOD on dementia can be confounded by various factors, including lifestyle and medical conditions.^29^ Other sociodemographic differences, including education level and employment status, were further found between EOD and LOD,^37^ which might also affect neurodegenerative prognosis. Further clarification of those factors could shed light on a potential pathway for dementia prevention in LOD.

Furthermore, this study found that deficiency in verbal reasoning and visuospatial memory can play a mediative role in the progression from LOD to dementia. Previous studies have suggested that decreased working memory could mediate between LLD and neuropsychological deficits.^38^ Despite that, the present study could raise awareness of the importance of verbal reasoning in progression to major cognitive impairment. One study found a mediative effect of reasoning between depressive symptoms and problem-solving ability in late life, suggesting that reasoning could be associated with loss of complex executive functioning.^39^ However, cognitive decline in this case accounted for a limited degree of mediation following full adjustment. This implies that the trajectory of cognitive decline over late life, rather than cognitive impairment at earlier stages, may be more involved in the progression to dementia in LLD.

Additionally, the present study noted that ELD showed better performance in numeric memory than healthy individuals and other groups with depression history. One potential explanation is that participants with only depression in the early life stages responded better to antidepressant therapies, which prevented their later episode and benefited their cognitive functioning in later life. One recent study suggested that better treatment response predicted a lower risk of dementia incidence following discharge,^40^ which supports this theory. This highlights the role of antidepressant response between LLD and dementia progression; given the complexity of this relationship, a separate investigation focusing on treatment patterns and clinical outcomes in LOD is currently under way.

This study has several strengths. First, it was conducted using one of the largest cohorts available, providing a substantial sample size that allowed for detailed investigation of differences among groups with distinct depression histories. To better account for the imbalance of baseline characteristics among healthy controls and groups with depression history, the present study introduced IPW and survey-based modelling to compensate. Furthermore, we employed a rigorous approach to identifying patients with a depressive history, relying solely on clinical records, which prioritised specificity. This study thus effectively ‘combines the best of two worlds’, i.e. benefits from the advantages of both a large population cohort and detailed clinical record data. Additionally, the study evaluated both cross-sectional cognitive changes across several domains and major neurocognitive outcomes, allowing us to capture both neuropsychological alterations and subsequent dementia diagnoses.

Certain limitations of this study should also be noted. Although clinical records offer a rigorous means of ascertaining depression cases, clinician-led records could also be susceptible to errors and inaccuracies. In addition, minor depression and other subclinical mood conditions were not fully considered due to inconsistent clinical notes and coding. Moreover, the proportion of dementia cases in our analysis was slightly lower than that observed in the general population,^4^ which has been observed in previous UKB research.^17^ This could be due to the overlap between history of other major psychiatric conditions and dementia incidence. Data-cleaning of the present analysis identified 4723 cases of dementia incidence in participants with primary care linked data (despite age, *n* = 231 300; see Supplementary Fig. 1), yet 2275 cases (48.17%) were excluded due to major psychiatric conditions. Furthermore, UKB administered only PHQ-2, a self-reported questionnaire with two items, as a measure for mood disturbances at baseline; consequently, the effect of ongoing depressive episode on cognitive performance might have been underestimated. Finally, the exclusion of participants who developed depression during follow-up may have led to the omission of cases where depressive symptoms were early manifestations of dementia, potentially introducing bias in the estimation of dementia risk.

This study shows that late-onset depression is associated with increased risk of cognitive decline and dementia. Further studies should focus on clinical and neurobiological factors to better understand the mechanism of age at depression onset affecting cognitive impairment. Longitudinal cohorts, with repeated symptom assessments and clinical evaluations, are needed to compare subgroup classifications and validate findings across different methodological approaches, which could bring more profound clinical indications.

## Supporting information

Xue et al. supplementary material Xue et al. supplementary material

## Data Availability

This publication is based on research using data from UKB through the UKB Research Analysis Platform.

## Supplementary material

The supplementary material is available online at https://doi.org/10.1192/bjo.2026.10995
