# Supplementary material for: Late-onset depression predicts cognitive impairment and subsequent dementia among older adults with major depressive disorder: findings from UK Biobank and primary care linked data
Source: BJPsych Open. 2026 Mar 10;12(2):e88. doi: 10.1192/bjo.2026.10995 (PMC13107299; doi:10.1192/bjo.2026.10995)
Supplement: Xue et al. supplementary material [file S2056472426109958sup001.docx]

**Supplementary Materials**

[Appendix 1 UK Biobank primary care linked data 2](#_Toc206501376)

[Figure S1 UK Biobank Inclusion flowchart^5^ 3](#_Toc206501377)

[Table S1 Read codes for depression diagnosis in UK Biobank primary care linked data 4](#_Toc206501378)

[Figure S2 Diagram of sample grouping by clinical depression history^1^ 5](#_Toc206501379)

[Appendix 2 Neuropsychological tests for baseline cognitive functioning 6](#_Toc206501380)

[Table S2 Between-group comparisons for baseline characteristics^1^ (Difference, Standard Error) 8](#_Toc206501381)

[Table S3 Weighted mean and standardized mean differences of propensity scores for all groups at all levels of survey-design based modelling^1^ (Mean, Standard Deviation) 9](#_Toc206501382)

[Table S4 Factor loadings of principal component analysis for cognitive composite score 11](#_Toc206501383)

[Table S5 Survey-weighted generalized linear mixed modelling at all levels^1^ (Mean and Standard Error of coefficients) 12](#_Toc206501384)

[Table S6 Weighted mean and standardized mean differences of propensity scores for all groups at all levels of Cox regression modelling^1^ (Mean, Standard Deviation) 14](#_Toc206501385)

[Table S7 Cox regression modelling predicting dementia incidence at all levels (Hazard Ratio with 95% Confidence Interval) 15](#_Toc206501386)

[Table S8 Post-hoc pairwise comparison of relative hazard ratio for all groups at all levels of modelling^1^ 16](#_Toc206501387)

[Table S9 Summary of regression-based causal mediation analysis (with fluid intelligence and pairs matching as mediators, non-exponentiated) 17](#_Toc206501388)

Appendix 1 UK Biobank primary care linked data

The UK Biobank (UKB) primary care linked data provide a valuable longitudinal resource capturing healthcare interactions at the general practice level for a subset of UKB participants. This interim release includes data from approximately 230,000 participants—roughly 45% of the UKB cohort—whose records were obtained through linkage with general practices across England, Scotland, and Wales. The database offers information on a wide range of records, including diagnoses, symptoms, procedures, laboratory results, immunisations, and prescriptions. These data are recorded by healthcare professionals such as general practitioners and practice nurses.

Clinical information is coded using Read version 2 (Read v2) or Clinical Terms Version 3 (CTV3, also known as Read v3), depending on the data provider and region. Prescription information is captured using Read codes, British National Formulary (BNF) codes, or the Dictionary of Medicines and Devices (dm+d), with variations in coding format and completeness across regions.

Data are provided with minimal curation to preserve their original structure and avoid introducing bias. Additionally, dates in the dataset are occasionally modified for participant confidentiality. Despite these limitations, the UKB primary care data offer an unprecedented opportunity for studying disease trajectories, drug exposure, and health outcomes over time in a large, well-characterized population.

Details can be found at <https://biobank.ndph.ox.ac.uk/showcase/refer.cgi?id=591>.

Figure S1 UK Biobank Inclusion flowchart^5^


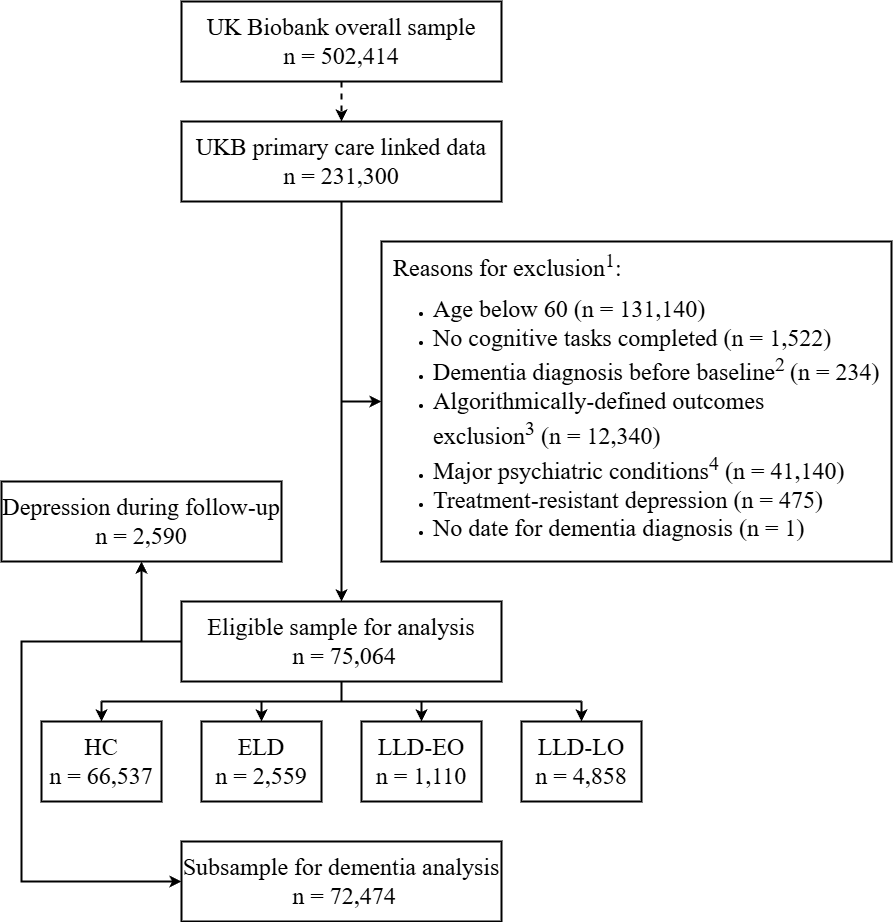


^1^: Numbers of participants excluded for each reason is not exclusive to each other.

^2^: Participants with dementia before or within one year after the assessment centre visit were excluded.

^3^: Algorithmically-defined outcomes for exclusion include end-stage renal disease, motor neurone disease, stroke, and all-cause parkinsonism.

^4^: Major psychiatric conditions for exclusion include parkinsonism, organic mental disorders, delirium, mental disorders due to physical disease, mental and behavioural disorders due to substance use, schizophrenia and psychotic disorders, and bipolar affective disorders.

^5^HC: Healthy controls; ELD: Early-life depression, LLD-EO; Late-life depression with early onset; LLD-LO: Late-life depression with late onset

Table S1 Read codes for depression diagnosis in UK Biobank primary care linked data

|  | Read_v2 | | | | Read_CTV3 | | | |
| --- | --- | --- | --- | --- | --- | --- | --- | --- |
| Diagnostic codes for depression | 12K8.  62T1.  E11..  E112.  E1120  E1121  E1122  E1123  E1124  E112z  E113.  E1130  E1131  E1132 | E1133  E1134  E1137  E113z  E118.  E11y2  E11z2  E130.  E135.  E204.  E2B..  E2B0.  E2B1.  Eu32. | Eu320  Eu321  Eu322  Eu323  Eu324  Eu325  Eu326  Eu327  Eu328  Eu329  Eu32A  Eu32B  Eu32y | Eu32z  Eu33.  Eu330  Eu331  Eu332  Eu333  Eu334  Eu33y  Eu33z  Eu341  Eu3y1  Eu530  R007z | 62T1.  E11..  E112.  E1120  E1121  E1122  E1123  E1124  E112z  E113.  E1130  E1131  E1132  E1133  E1134  E1137  E113z  E11y2  E130.  E204.  E2112  E2B..  E2B0.  E2B1.  Eu320  Eu321  Eu322  Eu323 | Eu32y  Eu32z  Eu33.  Eu330  Eu331  Eu332  Eu333  Eu334  Eu33y  Eu33z  Eu3y1  Eu530  R007z  X00SO  X00SQ  X00SR  X00SS  X00SU  X00Sb  X40Dl  X40Dm  X761L  X7755  X78x6  XE1Y0  XE1Y1  XE1YC  XE1ZY | XE1ZZ  XE1Za  XE1Zb  XE1Zc  XE1Zd  XE1Ze  XE1Zf  XE1aS  XE1aY  XM1GC  XSEGJ  XSGok  XSGol  XSGom  XSGon  Xa0wV  XaB9J  XaCHr  XaCHs  XaCIs  XaCIt  XaCIu  XaX53  XaX54  XaY2C  Xaeft  XagU1  XagUK | .62T1  .E221  .E222  .E234  .E35.  .E478  .E4J5  .E4J7  .E4J8  .E4J9  .E4JB  .E4JC  .R07Z  12K8.  E118.  E11z2  E135.  Eu32.  Eu324  Eu325  Eu326  Eu327  Eu328  Eu329  Eu32A  Eu32B  Eu341  Xa0Rd |
| Non-diagnostic codes for depression | 1465.  212S.  9HA0. | 9HA1.  9k4..  9k40. | 9kQ..  E1125  E1126 | E1135  E1136 | 1465.  E1125  E1126  E1135  E1136  XaJWh  XaJWi | XaK9p  XaKAK  XaLG0  XaNg1  XaPKm  .1465 | .2257  .9HA0  .9HA1  .9k4.  .9k40  .9kQ. | 212S.  9HA0.  9HA1.  9k4..  9k40.  9kQ.. |

Figure S2 Diagram of sample grouping by clinical depression history^1^


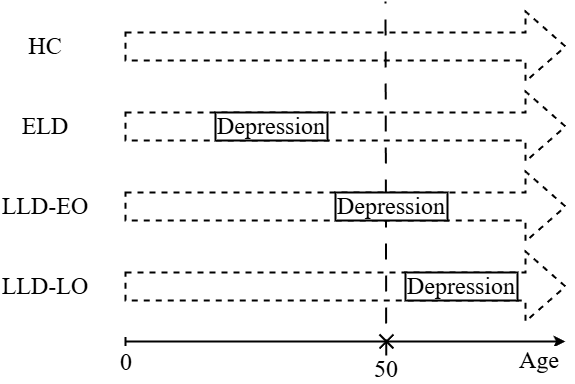


^1^HC: Healthy controls; ELD: Early-life depression, LLD-EO; Late-life depression with early onset; LLD-LO: Late-life depression with late onset. Participants were classified as HC if they had no lifetime depressive episodes, or ELD if all episodes were before age50, or LLD-EO if their episodes started before 50 and ended after 50, or LLD-LO if all their episodes started after age 50.

Appendix 2 Neuropsychological tests for baseline cognitive functioning

five tests were administered at baseline at the UK Biobank Assessment Centre. Participants were required to respond to the assigned tasks or questions via a touchscreen interface during their baseline visit.

S2.1 Reaction Time (Snap) Test

The Reaction Time (Snap) test was administered using a touch-screen computer and a button box specifically designed for participant interaction. Participants were seated and instructed to rest their dominant hand over the button box. On each trial, two visual cards were displayed side-by-side on the screen. Participants were asked to press the button as quickly as possible whenever the symbols on the two cards matched. Each participant completed 12 pairs of card trials. The test did not include an option to abandon once started, and the first five rounds were considered training trials. The primary outcome measure was mean reaction time to correct matches, derived by averaging the response times for all valid match trials (excluding training rounds).

S2.2 Fluid Intelligence Test

Fluid intelligence reflects an individual's ability to reason and solve novel problems, independent of acquired knowledge. In this test, participants were given a maximum of two minutes to answer as many questions as possible. A brief on-screen instruction encouraged participants to work quickly, skip difficult items, and avoid spending too long on any single question. Questions were automatically presented in sequence, and participants responded using the touchscreen. If the full two-minute limit elapsed, the test ended automatically. Participants could opt out before beginning, in which case the test was skipped. The questions involved a variety of reasoning types, including arithmetic, verbal analogies, deductive reasoning, and pattern recognition (e.g., completing sequences, identifying relationships). Answer choices included plausible distractors as well as options such as “Do not know” or “Prefer not to answer.” Performance on this task was measured by the number of correct answers completed within the two-minute period.

S2.3 Pairs Matching (Visuospatial Memory) Test

The task assessed visuospatial memory by challenging participants to recall the positions of matching symbol cards. Participants were guided through a brief video demonstration before commencing the task, and used the touchscreen to interact with a grid of visual cards. Two rounds were presented in the main recruitment phase. In the first round, three pairs (six cards) were displayed randomly on the screen for three seconds, after which the cards turned face down. The participant was then asked to identify matching pairs by selecting cards on the screen. In the second round, six pairs (twelve cards) were shown for five seconds before being hidden. Participants attempted to match all pairs with as few errors as possible. There was no time limit for responding, and the card layout was randomly generated in each round. The task could be skipped or abandoned via a designated button. The primary outcome measure was the incorrect matches of all pairs, the more of which indicated worse responses.

S2.4 Numeric Memory Test

This task assessed working memory by presenting participants with a number of increasing length to recall and re-enter. The test began with a 2-digit number, displayed for a duration based on its length (2000 ms plus 500 ms per digit). During display, the number pad was disabled. After the number disappeared and a short delay (3000 ms), the participant was prompted to re-enter the number using the on-screen keypad. If entered correctly, the next number increased by one digit. This process continued up to a maximum of 12 digits. If the number was 2 digits, the test ended after 5 consecutive incorrect responses; for numbers with 3 or more digits, it ended after 2 consecutive errors. Digits were pseudo-randomised so no digit repeated immediately or after a single intervening digit. The primary outcome measure was the maximum number of digits correctly recalled. Outcomes that were outside five standard deviations of the sample were nullified.

S2.5 Prospective Memory Test

The Prospective Memory was the first and last of a series of cognitive tasks completed during the visit. The task was designed to evaluate prospective memory—the ability to remember to perform an intended action after a delay. The task consisted of two parts. Before the other cognitive tests began, participants were shown an instruction that at the end of the session, when presented with four coloured shapes, they would be told to select the Blue Square but should actually select the Orange Circle. This was followed by other cognitive tasks, creating a delay between instruction and response. After completing the preceding tests, participants were presented with four coloured shapes again and instructed again to touch the Blue Square. Participants who selected the Blue Square were prompted to recall the earlier instruction to select the Orange Circle instead. The test ended once they made a selection other than the Blue Square. The primary outcome measure was their correct response as a binary indicator.

Further details can be found at <https://biobank.ndph.ox.ac.uk/showcase/>.

Table S2 Between-group comparisons for baseline characteristics^1^ (Difference, Standard Error)

| Pairwise comparison | Age | Sex, Male | BMI | Education, College | TDI | MET | Smoking, Smokers | Alcohol use | Physical Diseases | PHQ-2 | Age at onset^2^ | Multiple episodes | Dementia incidence |
| --- | --- | --- | --- | --- | --- | --- | --- | --- | --- | --- | --- | --- | --- |
| HC – LO | -0.03 (0.04) | **χ^2^_(3)_ =711.41,**  ***p* < .001** | **-0.69 (0.07)** | **χ^2^_(3)_ =24.39,**  ***p* < .001** | **-0.26 (0.04)** | 0.03  (0.02) | **χ^2^_(3)_ =11.33,**  ***p* = .010** | **-0.30 (0.02)** | **χ^2^_(3)_ =41.50,**  ***p* < .001** | **-0.57 (0.01)** | - | - | ***χ^2^*_(3)_ = 22.93, *p* < .001** |
| HC – ELD | **0.58 (0.06)** |  | **-0.26 (0.09)** |  | -0.10 (0.06) | 0.06  (0.02) |  | **-0.18 (0.03)** |  | **-0.30 (0.02)** | - | **χ^2^_(3)_ =2863.3,**  ***p* < .001** |  |
| HC – EO | **0.65 (0.09)** |  | **-0.74 (0.13)** |  | **-0.26 (0.08)** | 0.06  (0.04) |  | **-0.41 (0.05)** |  | **-0.88 (0.03)** | - |  | Dementia Types |
| LO – ELD | **0.60 (0.07)** |  | **0.43 (0.11)** |  | 0.16 (0.07) | 0.02  (0.03) |  | **0.13 (0.04)** |  | **0.27 (0.02)** | - |  |  |
| LO – EO | **0.68 (0.09)** |  | **-0.05 (0.15)** |  | -0.01 (0.09) | 0.02  (0.04) |  | -0.11 (0.05) |  | **-0.31 (0.03)** | **0.61 (0.15)** |  | *χ^2^*_(3)_ =0.90, *p* = .99 |
| ELD – EO | 0.08 (0.10) |  | **-0.48 (0.16)** |  | -0.17 (0.10) | -0.01  (0.04) |  | **-0.23 (0.06)** |  | **-0.58 (0.03)** | **-1.77 (0.34)** |  |  |

^1^HC: Healthy Controls; ELD: Early-Life Depression; EO: (Late-Life Depression) Early Onset; LO: (Late-Life Depression) Late Onset; BMI: Body Mass Index; TDI: Townsend Deprivation Index; MET: Metabolic Equivalent Task score; PHQ-2: Patient Health Questionnaire-2. Difference and standard error presented in bold in the table suggested significant differences than zero after Bonferroni adjustment for multiple testing.

^2^Age at first depressive episode was compared between ELD and EO groups, while age at last depressive episode was compared between LO and EO groups.

Table S3 Weighted mean and standardized mean differences of propensity scores for all groups at all levels of survey-design based modelling^1^ (Mean, Standard Deviation)

| Confounders | HC | LO | ELD | EO | SMD |
| --- | --- | --- | --- | --- | --- |
| Level 1 | | | | | |
| Weighted N | 75064.33 | 75063.35 | 74932.76 | 74933.96 | - |
| Age | 64.52  (2.85) | 64.52  (2.83) | 64.50  (2.85) | 64.49  (2.80) | 0.007 |
| Sex | 0.44  (0.50) | 0.44  (0.50) | 0.43  (0.50) | 0.44  (0.50) | 0.004 |
| Level 2 | | | | | |
| Weighted N | 54640.14 | 54547.88 | 54596.79 | 54551.03 | - |
| Age | 64.33  (2.82) | 64.35  (2.82) | 64.32  (2.83) | 64.29  (2.74) | 0.011 |
| Sex | 0.45  (0.50) | 0.45  (0.50) | 0.45  (0.50) | 0.44  0.50) | 0.004 |
| BMI | 27.25  (4.36) | 27.30  (4.26) | 27.21  (4.44) | 27.16  (4.48) | 0.018 |
| TDI | -2.04  (2.62) | -2.01  (2.62) | -2.05  (2.62) | -1.97  (2.73) | 0.016 |
| MET | 7.47  (1.02) | 7.46  (1.06) | 7.47  (1.03) | 7.49  (1.08) | 0.014 |
| Education | 0.37  (0.48) | 0.37  (0.48) | 0.38  (0.48) | 0.37  (0.48) | 0.008 |
| Level 3 | | | | | |
| Weighted N | 54481.91 | 54389.66 | 54422.33 | 54611.19 |  |
| Age | 64.33  (2.82) | 64.35  (2.82) | 64.31  (2.83) | 64.28  (2.72) | 0.013 |
| Sex | 0.45  (0.50) | 0.45  (0.50) | 0.45  (0.50) | 0.45  (0.50) | 0.003 |
| BMI | 27.25  (4.36) | 27.31  (4.26) | 27.20  (4.44) | 27.14  (4.45) | 0.021 |
| TDI | -2.04  (2.62) | -2.02  (2.61) | -2.05  (2.61) | -1.96  (2.71) | 0.019 |
| MET | 7.47  (1.02) | 7.46  (1.06) | 7.47  (1.02) | 7.49  (1.08) | 0.013 |
| Education | 0.37  (0.48) | 0.37  (0.48) | 0.38  (0.48) | 0.37  (0.48) | 0.006 |
| Alcohol use | 2.79  (1.53) | 2.79  (1.52) | 2.78  (1.53) | 2.75  (1.57) | 0.013 |
| Smoking | 0.42  (0.49) | 0.42  (0.49) | 0.42  (0.49) | 0.41  (0.49) | 0.011 |

^1^HC: Healthy Controls; ELD: Early-Life Depression; EO: (Late-Life Depression) Early Onset; LO: (Late-Life Depression) Late Onset; SMD: Standard Mean Difference; BMI: Body Mass Index; TDI: Townsend Deprivation Index; MET: Metabolic Equivalent Task score. Levels of adjustment. Level 0: Unadjusted; Level 1: Adjusted for age and sex; Level 2: Adjusted for age, sex, BMI, PHQ-2 score, education, MET and TDI; Level 3: Adjusted for age, sex, BMI, PHQ-2 score, education, MET, TDI, smoking and alcohol use status.

Table S4 Factor loadings of principal component analysis for cognitive composite score

| Measures | PC1 | PC2 | PC3 | PC4 |
| --- | --- | --- | --- | --- |
| Reaction time | 0.40 | -0.84 | 0.36 | -0.02 |
| Fluid intelligence | 0.54 | 0.29 | 0.10 | 0.73 |
| Pairs matching | 0.31 | -0.23 | -0.90 | -0.09 |
| Numeric memory | 0.47 | 0.35 | 0.22 | -0.67 |
| Prospective memory | 0.47 | 0.17 | -0.04 | -0.09 |
| Variance explained (%) | 36.47 | 19.79 | 16.58 | 15.54 |

Table S5 Survey-weighted generalized linear mixed modelling at all levels^1^ (Mean and Standard Error of coefficients)

| Variables | Cognitive composite | Reaction time | Fluid intelligence | Pairs matching | Numeric memory | Prospective memory |
| --- | --- | --- | --- | --- | --- | --- |
| Level 0 | | | | | | |
| ELD | 0.06  (0.06) | 0.02  (0.02) | 0.02  (0.03) | 0.04  (0.02) | **0.13****  **(0.05)** | 0.00  (0.08) |
| LLD-EO | -0.11  (0.10) | **0.11*****  **(0.03)** | -0.04  (0.05) | -0.02  (0.03) | -0.05  (0.07) | 0.11  (0.11) |
| LLD-LO | **-0.17*****  **(0.05)** | **0.08*****  **(0.00)** | **-0.17*****  **(0.02)** | **0.08*****  **(0.01)** | -0.07  (0.04) | **-0.17****  **(0.05)** |
| Level 1 | | | | | | |
| ELD | 0.07  (0.07) | 0.01  (0.02) | 0.04  (0.04) | **0.05****  **(0.02)** | **0.17*****  **(0.04)** | -0.03  (0.08) |
| LLD-EO | -0.02  (0.11) | **0.09****  **(0.03)** | -0.03  (0.05) | 0.02  (0.04) | -0.00  (0.06) | 0.11  (0.12) |
| LLD-LO | **-0.17*****  **(0.05)** | **0.06*****  **(0.02)** | **-0.16*****  **(0.02)** | **0.08*****  **(0.01)** | -0.06  (0.04) | **-0.17****  **(0.05)** |
| Age | **-0.06*****  **(0.01)** | **0.04*****  **(0.00)** | **-0.04*****  **(0.01)** | **0.03*****  **(0.00)** | **-0.02***  **(0.01**) | **-0.06*****  **(0.01)** |
| Sex | **0.22****  **(0.08)** | **-0.20*****  **(0.02)** | **0.15*****  **(0.04)** | -0.00  (0.02) | **0.18*****  **(0.04)** | 0.07  (0.08) |
| Level 2 | | | | | | |
| ELD | 0.12  (0.08) | -0.00  (0.03) | **0.08***  **(0.04)** | 0.04  (0.05) | **0.18*****  **(0.04)** | -0.04  (0.11) |
| LLD-EO | 0.08  (0.14) | **0.09***  **(0.04)** | 0.06  (0.07) | 0.04  (0.05) | -0.01  (0.07) | **0.44***  **(0.20)** |
| LLD-LO | -0.09  (0.06) | 0.03  (0.02) | **-0.08***  **(0.03)** | **0.06****  **(0.02)** | -0.03  (0.04) | -0.13  (0.08) |
| Age | **-0.03***  **(0.01)** | **0.04*****  **(0.00)** | **-0.02***  **(0.00)** | **0.03*****  **(0.00)** | -0.01  (0.01) | -0.02  (0.02) |
| Sex | **0.33*****  **(0.08)** | **-0.24*****  **(0.03)** | **0.16*****  **(0.04)** | -0.00  (0.03) | **0.17*****  **(0.05)** | 0.14  (0.11) |
| PHQ-2 score | -0.07  (0.04) | **0.03***  **(0.01)** | -0.04  (0.02) | 0.02  (0.02) | -0.02  (0.02) | **-0.10***  **(0.05)** |
| BMI | **-0.02***  **(0.01)** | 0.00  (0.00) | -0.01  (0.00) | **-0.01***  **(0.00)** | **-0.01****  **(0.01)** | -0.01  (0.01) |
| TDI | -0.03  (0.02) | **0.01****  **(0.00)** | -0.02  (0.01) | 0.00  (0.01) | -0.00  (0.01) | -0.02  (0.02) |
| MET | **-0.11****  **(0.04)** | 0.00  (0.01) | **-0.11*****  **(0.02)** | 0.00  (0.02) | **-0.05***  **(0.02)** | **-0.14***  **(0.06)** |
| Edu-cation | **0.47****  **(0.07)** | -0.05  (0.03) | **0.50*****  **(0.04)** | **-0.07***  **(0.03)** | **0.18*****  **(0.05)** | **0.36****  **(0.12)** |
| Level 3 | | | | | | |
| ELD | 0.10  (0.08) | -0.01  (0.03) | 0.07  (0.04) | 0.00  (0.03) | **0.18*****  **(0.04)** | -0.10  (0.12) |
| LLD-EO | -0.11  (0.08) | 0.08  (0.05) | 0.06  (0.07) | 0.02  (0.05) | 0.01  (0.08) | 0.34  (0.23) |
| LLD-LO | -0.11  (0.08) | 0.01  (0.03) | **-0.09***  **(0.04)** | **0.05***  **(0.02)** | -0.03  (0.05) | -0.20  (0.12) |
| Age | **-0.03****  **(0.01)** | **0.04*****  **(0.00)** | **-0.02***  **(0.01)** | **0.03*****  **(0.01)** | -0.00  (0.01) | -0.02  (0.02) |
| Sex | **0.26****  **(0.08)** | **-0.22*****  **(0.03)** | **0.11***  **(0.05)** | 0.02  (0.03) | **0.14****  **(0.05)** | 0.07  (0.12) |
| PHQ-2 score | -0.06  (0.04) | 0.03  (0.01) | **-0.04***  **(0.02)** | 0.02  (0.02) | -0.02  (0.02) | -0.09  (0.05) |
| BMI | -0.02  (0.01) | -0.00  (0.00) | -0.01  (0.00) | **-0.01***  **(0.00)** | **-0.01****  **(0.01)** | -0.01  (0.01) |
| TDI | -0.01  (0.02) | **0.01****  **(0.00)** | -0.01  (0.01) | 0.01  (0.01) | -0.00  (0.01) | -0.02  (0.02) |
| MET | **-0.12*****  **(0.04)** | 0.00  (0.01) | **-0.11*****  **(0.02)** | 0.00  (0.02) | **-0.06***  **(0.02)** | **-0.16****  **(0.06)** |
| Edu-cation | **0.45*****  **(0.09)** | -0.05  (0.03) | **0.47*****  **(0.04)** | **-0.07***  **(0.03)** | **0.16*****  **(0.05)** | **0.31***  **(0.12)** |
| Alcohol | **-0.07****  **(0.02)** | **0.02***  **(0.01)** | **-0.05*****  **(0.01)** | 0.00  (0.01) | **-0.03***  **(0.02)** | **-0.10****  **(0.04)** |
| Smoking | 0.06  (0.08) | -0.00  (0.03) | 0.01  (0.04) | **-0.10****  **(0.03)** | -0.04  (0.05) | **0.26***  **(0.12)** |
| Physical Diseases | 0.02  (0.09) | 0.02  (0.03) | -0.01  (0.05) | -0.0  (0.03) | 0.07  (0.05) | -0.15  (0.14) |
| Depression Treatment | 0.04  (0.09) | 0.02  (0.03) | 0.02  (0.05) | 0.04  (0.04) | 0.00  (0.06) | 0.13  (0.14) |

*: *p* < .05; **: *p* < .01; ***: *p* < .001

^1^ELD: Early-life depression, LLD-EO; Late-life depression with early onset; LLD-LO: Late-life depression with late onset. All groups with depression history were compared with depression-free Healthy Controls as baseline reference. BMI: Body Mass Index; TDI: Townsend Deprivation Index; MET: Metabolic Equivalent Task score; PHQ-2: Patient Health Questionnaire-2.

Levels of adjustment. Level 0: Unadjusted; Level 1: Adjusted for age and sex; Level 2: Adjusted for age, sex, BMI, PHQ-2 score, education, MET and TDI; Level 3: Adjusted for age, sex, BMI, PHQ-2 score, education, MET, TDI, smoking, alcohol use status, physical diseases and depression treatment.

Table S6 Weighted mean and standardized mean differences of propensity scores for all groups at all levels of Cox regression modelling^1^ (Mean, Standard Deviation)

| Confounders | HC | LO | ELD | EO | SMD |
| --- | --- | --- | --- | --- | --- |
| Level 1 | | | | | |
| Weighted N | 72474.28 | 72474.42 | 72362.79 | 72337.75 | - |
| Age | 64.53  (2.84) | 64.53  (2.83) | 64.51  (2.84) | 64.48  (2.71) | 0.010 |
| Sex | 0.44  (0.50) | 0.44  (0.50) | 0.44  (0.50) | 0.44  (0.50) | 0.003 |
| Level 2 | | | | | |
| Weighted N | 52896.50 | 52810.15 | 52850.80 | 52603.71 | - |
| Age | 64.33  (2.82) | 64.35  (2.81) | 64.33  (2.83) | 64.25  (2.66) | 0.018 |
| Sex | 0.45  (0.50) | 0.45  (0.50) | 0.45  (0.50) | 0.44  0.50) | 0.009 |
| BMI | 27.23  (4.34) | 27.29  (4.22) | 27.20  (4.43) | 27.17  (4.45) | 0.015 |
| TDI | -2.04  (2.61) | -2.03  (2.63) | -2.06  (2.61) | -1.95  (2.77) | 0.022 |
| MET | 7.47  (1.02) | 7.46  (1.06) | 7.47  (1.02) | 7.48  (1.09) | 0.009 |
| Education | 0.37  (0.48) | 0.37  (0.48) | 0.37  (0.48) | 0.37  (0.48) | 0.006 |
| Level 3 | | | | | |
| Weighted N | 52748.34 | 52627.56 | 52688.57 | 52635.91 |  |
| Age | 64.33  (2.82) | 64.35  (2.82) | 64.33  (2.84) | 64.24  (2.63) | 0.022 |
| Sex | 0.45  (0.50) | 0.45  (0.50) | 0.45  (0.50) | 0.45  (0.50) | 0.007 |
| BMI | 27.23  (4.34) | 27.30  (4.22) | 27.18  (4.42) | 27.16  (4.40) | 0.019 |
| TDI | -2.05  (2.61) | -2.04  (2.61) | -2.06  (2.60) | -1.94  (2.76) | 0.024 |
| MET | 7.47  (1.02) | 7.46  (1.05) | 7.47  (1.02) | 7.48  (1.09) | 0.005 |
| Education | 0.37  (0.48) | 0.37  (0.48) | 0.38  (0.48) | 0.38  (0.49) | 0.007 |
| Alcohol use | 2.78  (1.52) | 2.78  (1.52) | 2.77  (1.53) | 2.74  (1.58) | 0.014 |
| Smoking | 0.42  (0.49) | 0.42  (0.49) | 0.42  (0.49) | 0.41  (0.49) | 0.009 |

^1^HC: Healthy Controls; ELD: Early-Life Depression; EO: (Late-Life Depression) Early Onset; LO: (Late-Life Depression) Late Onset; SMD: Standard Mean Difference; BMI: Body Mass Index; TDI: Townsend Deprivation Index; MET: Metabolic Equivalent Task score. Levels of adjustment. Level 0: Unadjusted; Level 1: Adjusted for age and sex; Level 2: Adjusted for age, sex, BMI, PHQ-2 score, education, MET and TDI; Level 3: Adjusted for age, sex, BMI, PHQ-2 score, education, MET, TDI, smoking and alcohol use status.

Table S7 Cox regression modelling predicting dementia incidence at all levels (Hazard Ratio with 95% Confidence Interval)

| Variables | Level 0 | Level 1 | Level 2 | Level 3 |
| --- | --- | --- | --- | --- |
| ELD | 1.07  [0.80, 1.44] | 1.25  [0.91, 1.72] | 0.91  [0.53, 1.59] | 0.85  [0.48, 1.51] |
| EO | 1.23  [0.77, 1.96] | **1.77***  **[1.02, 3.07]** | 1.58  [0.71, 3.5] | 1.72  [0.81, 3.67] |
| LO | **1.49*****  **[1.22, 1.82]** | **1.52*****  **[1.24, 1.86]** | **1.64****  **[1.15, 2.33]** | **1.57***  **[1.10, 2.26]** |
| Age | - | **1.31*****  **[1.22, 1.40]** | **1.37*****  **[1.26, 1.49]** | **1.329*****  **[1.27, 1.51]** |
| Sex | - | 1.32  [0.91, 1.91] | **1.94***  **[1.13, 3.30]** | **1.39***  **[0.88, 2.20]** |
| PHQ-2 score | - | - | 1.27  [1.00, 1.63] | **1.33***  **[1.06, 1.66]** |
| BMI | - | - | 0.94  [0.87, 1.01] | 0.94  [0.87, 1.01] |
| TDI | - | - | 1.06  [0.97, 1.17] | 1.05  [0.96, 1.18] |
| MET | - | - | 1.02  [0.76, 1.38] | 1.05  [0.76, 1.45] |
| Education | - | - | 1.08  [0.59, 1.99] | 1.07  [0.59, 1.93] |
| Alcohol | - | - | - | 1.05  [0.88, 1.24] |
| Smoking | - | - | - | 1.27  [0.74, 2.16] |
| Physical Diseases |  |  |  | 0.91  [0.47, 1.76] |
| Depression Treatment |  |  |  | 1.25  [0.60, 2.63] |

*: *p* < .05; **: *p* < .01; ***: *p* < .001

^1^ELD: Early-life depression, EO; Late-life depression with early onset; LO: Late-life depression with late onset. All groups with depression history were compared with depression-free Healthy Controls as baseline reference. BMI: Body Mass Index; TDI: Townsend Deprivation Index; MET: Metabolic Equivalent Task score; PHQ-2: Patient Health Questionnaire-2.

Levels of adjustment. Level 0: Unadjusted; Level 1: Adjusted for age and sex; Level 2: Adjusted for age, sex, BMI, PHQ-2 score, education, MET and TDI; Level 3: Adjusted for age, sex, BMI, PHQ-2 score, education, MET, TDI, smoking, alcohol use status, physical diseases and depression treatment.

Table S8 Post-hoc pairwise comparison of relative hazard ratio for all groups at all levels of modelling^1^

| Pairwise comparison | Level 0 | Level 1 | Level 2 | Level 3 |
| --- | --- | --- | --- | --- |
| HC / LO | **0.67*****  **(0.07)** | **0.66*****  **(0.07)** | **0.61***  **(0.11)** | 0.63  (0.12) |
| HC / ELD | 0.94  (0.14) | 0.80  (0.12) | 1.09  (0.30) | 1.17  (0.34) |
| HC / EO | 0.81  (0.18) | 0.56  (0.16) | 0.63  (0.26) | 0.58  (0.22) |
| LO / ELD | 1.39  (0.25) | 1.22  (0.23) | 1.79  (0.57) | 1.85  (0.60) |
| LO / EO | 1.21  (0.31) | 0.86  (0.25) | 1.04  (0.43) | 0.91  (0.37) |
| ELD / EO | 0.87  (0.24) | 0.70  (0.22) | 0.58  (0.28) | 0.49  (0.24) |

*: *p* < .05; **: *p* < .01; ***: *p* < .001

^1^HC: Healthy Controls; ELD: Early-Life Depression; EO: (Late-Life Depression) Early Onset; LO: (Late-Life Depression) Late Onset. Relative hazard ratio and standard error were after Bonferroni adjustment for multiple testing.

Table S9 Summary of regression-based causal mediation analysis (with fluid intelligence and pairs matching as mediators, non-exponentiated)

| Effects | Fluid intelligence | | Pairs matching | |
| --- | --- | --- | --- | --- |
|  | Estimate | *p* value | Estimate | *p* value |
| Average causal mediation effect | 3.14 × 10^-4^ | <.001 | 1.29 × 10^-4^ | .005 |
| Average direct effect | 9.78 × 10^-3^ | <.001 | 8.91 × 10^-3^ | <.001 |
| Average proportion mediated (%) | 3.09 | <.001 | 1.41 | .005 |
